# Changes in Child and Youth Mental Health Following the Return To In-Person Learning Post-COVID-19 Pandemic

**DOI:** 10.1007/s11920-025-01642-4

**Published:** 2025-10-07

**Authors:** Erika Felix, Jennifer Greif Green

**Affiliations:** 1 Department of Counseling, Clinical, and School Psychology, Gevirtz Graduate School of Education, University of California Santa Barbara, Santa Barbara, CA 93101 USA; 2 Wheelock College of Education & Human Development, Boston University, Boston, USA

**Keywords:** COVID-19 pandemic, Child, Adolescent, Mental health, Schools, Disaster

## Abstract

**Purpose of Review:**

Changes in youth mental health during the pandemic have been well documented globally, but research on how mental health changed when schools returned to in-person learning is just emerging. This review summarizes the available global research on child and youth mental health following school reopening for in-person learning.

**Recent Findings:**

Results varied by the mental health indicator being assessed and by subgroups of children and youth, with age-related differences, and possible gender-related influences. Some modifiable risk and protective factors examined included time spent on homework; internet and social media use; physical activity; communication/conflict with others; optimism; social relationships with family, teacher and peers; parental mental health; and inconsistent discipline.

**Summary:**

Some youth fared better when schools reopened in-person, but for others mental health challenges persisted. Mental health services shifted during the height of the pandemic, and some supports are no longer available. Continued monitoring is needed to help with recovery and resilience.

## Introduction

In January of 2020, the first case of severe acute respiratory syndrome coronavirus 2 (SARS‑CoV‑2 or COVID-19) was identified in the United States (U.S.) [1]. By spring of 2020, almost all pre-Kindergarten to grade 12 schools in the U.S. were closed to prevent the transmission of the virus [2]. Although most schools reopened in fall of 2020 with a combination of in-person, virtual learning, and hybrid learning, there continued to be both brief and extended periods of time when schools were closed to in-person learning in U.S. communities, largely as a reaction to local transmission of the virus [2]. Globally, schools and communities struggled to contain the pandemic, often engaging in closures or shifts to remote learning in schools and other community-serving settings [3]. The massive scale of the COVID-19 pandemic and associated losses, as well as shifts in services provided by community organizations, including schools, offer an opportunity to consider how large-scale disasters impact youth mental health and mental health services.

Significant concerns arose about the mental health of youth during the pandemic, which were often described in the context of school building closure and associated loss of learning opportunities [4]. In general, the closure of school buildings and shift to virtual and hybrid learning caused substantial disruption to student learning [5]. Although it has been difficult to estimate the magnitude of the initial impact, or subsequent recovery, a meta-analysis of studies comparing student achievement data prior to and during the pandemic found a statistically significant decline in academic achievement worldwide [6]. Similarly, data from 72 countries involved in the Program for International Student Assessment study reported a decline in mathematics scores equivalent to the loss of about 7 months of learning from 2018 to 2022. [7] However, early evidence indicated that the pandemic and associated suspension of in-person learning had a differential impact on youth, often exacerbating existing inequities. For example, Jakubowski et al. [7] found greater learning loss reported in countries with longer periods of time when students did not have access to in-person learning. In the U.S., data suggest that inequities in learning outcomes were associated with differential access to social and economic resources, including youth access to learning spaces and digital technology, parental access to remote work opportunities, and continuity in receipt of academic and social-emotional supports – all of which disproportionately impacted students with disabilities, those living in lower income communities, and racially minoritized families [8]. 

In addition to documented learning loss from school building closures, there is evidence that children and youth experienced worsening mental health during the pandemic and related suspension of in-person schooling. A body of research comparing the mental health of youth prior to the pandemic, to after the pandemic’s start, consistently found an increase of mental health symptoms [9–11]. However, the magnitude of these increases were relatively small and followed a rising trend that began pre-pandemic [12]. In addition, these published studies focused almost entirely on changes in mental health occurring during school building closure, or partial reopening and remote learning. Very few studies have presented a longer-term perspective on trends in mental health following the return to in-person learning. As Wade et al., [13] describe, longer-term longitudinal data are necessary to understand the continuing impact of the pandemic, as well as to explain the different ways that pandemic-related stressors impacted different youth. The following sections review the research to-date on the association between school building reopening following the COVID-19 pandemic restrictions and child and youth mental health over time.

## Return To In-Person Learning and Youth Mental Health

Based on knowledge from disaster mental health research on children and youth, multiple trajectories of adaptation would be expected, with resilience, recovery, and chronic impairment patterns [14]. Multiple aspects of the recovery environment will affect child and youth adaptations, with schools playing a critical role [15]. Here we describe the results of several studies conducted globally to identify trends in youth mental health following school return to in-person learning.

Results are nuanced, revealing different patterns among different groups of students. A nationwide, cross-sectional study of government schools in Malaysia conducted in 2022 tested optimistic and pessimistic views of what a return to in-person school would entail [16]. They found that the majority of primary (72%) and secondary school (59%) students were happy to be back at school in-person and reported fewer education-related worries and negative emotions. However, a significant minority of students reported that even after returning to school in-person, they did not learn more, were struggling to catch up, and were concerned about learning loss. There were age-related differences, with secondary school students being less favorable about the return to in-person school and less happy following reopening compared to primary school students. However, they were also less likely to be absent than primary school students.

A short-term longitudinal study in Shanghai, China looked at adolescent mental health before (April, 2020) and three months after (July, 2020) school building reopening in that region [17]. Although overall, adolescents reported fewer symptoms of anxiety, depression, and anger three months after a return to in-person school, this depended on perceived levels of academic stress and whether or not the student had a performance or mastery academic orientation, with higher mastery orientation buffering the impact of academic stress on mental health. A study in Hong Kong collected two waves of cross-sectional data from parents of children with special educational needs, one while school buildings were closed in April 2020 and again a few months after buildings reopened (July-October 2021) [18]. They found that after buildings reopened, preschoolers with special educational needs had fewer emotional and conduct difficulties, but adolescents had more conduct difficulties, and children 6–11 years old had worse quality of life, per parents.

In Finland, researchers conducted a repeated cross-sectional, national population-based study of secondary school students (ages 13–20 years old) looking at academic years 2015–2023 [19]. They found that the proportion of students above clinical cut-offs for generalized anxiety, social anxiety, and depression increased from pre-pandemic to during pandemic and remained higher in 2023 across all demographic groups assessed. Overall mental well-being scores decreased for all student groups between 2021 and 2023, but there were also reductions in feelings of loneliness across all groups as well. Finally, disordered eating increased among girls and younger boys, and suicidality increased among girls but not boys.

A comprehensive population-based study from the Netherlands examined changes in mental health in both the general population of children and youth, and within a clinical sample, from pre-pandemic to March/April of 2023 [20]. Data collection time points were mapped onto the patterns of schools closing and re-opening during the pandemic, and two timepoints post-pandemic when schools remained open. In both the general population sample and clinical sample, parents reported significantly higher levels of internalizing problems compared to pre-pandemic levels, but not for externalizing behavior problems. Likewise, their child-report data for both clinical and general population samples showed the greatest increases in anxiety and depression, followed by changes in general health, sleep, and anger. For the population sample, they note that current mental health symptoms are higher than pre-pandemic levels, whereas for the clinical sample, data collection was only during and after the pandemic. In examining the data for after schools permanently re-opened, the trends varied by mental health indicator, but remained elevated compared to pre-pandemic levels (community sample) or early pandemic (clinical sample).

A Canadian study of children’s mental health during (3 timepoints) and after the pandemic (one timepoint) that accounted for the effects of age, because odds of having mental health symptoms increase with age, found a curvilinear pattern for depression, anxiety, hyperactivity and inattention symptoms [21]. Highest levels of symptoms were at the start of the pandemic and then decreased over time, but increased after schools returned to in-person instruction. Unlike the Finnish [19] study, they did not tend to find sociodemographics modifying the trajectories of mental health symptoms [21]. In Iceland, a repeated, cross-sectional, population-based study of adolescents from 2016 to 2023 found that depression, anxiety, and hostility all increased in 2021, compared to pre-pandemic levels [22]. However, depression improved from 2021 to 2023, suggesting some post-pandemic recovery.

An Australian study with two pre-pandemic, one during pandemic, and one timepoint following schools reopening found an increase in depression symptoms, internalizing symptoms, feelings of isolation, and a decrease in well-being compared to pre-pandemic that remained elevated following school return to in-person learning [23]. There was no change in externalizing symptoms. They then examined trajectories of symptoms based on levels of symptoms from pre-pandemic, finding the smallest increases in symptoms among those with the highest pre-pandemic symptoms, and the largest increase for those with the lowest pre-pandemic symptoms. Gender also influenced results, with females reporting more symptoms and lower levels well-being than males. Notably, the authors assessed attitudes about being alone, finding that those with more negative attitudes about being alone had higher rates of depression, while those with positive attitudes about being alone reported higher levels of well-being.

Within the U.S., data from the national Youth Risk Behavior Surveillance Survey (YRBS) indicate that youth reported an increase in depressive symptoms and suicidality from 2013 to 2023 overall, with a particularly sharp increase among females from 2019 to 2021 [24]. A subsequent decline in these rates among females, from 2021 to 2023, indicates some improvement in youth-reported mental health. A dissertation study using repeated cross-sectional data from 40 schools in California found that 6th-8th grade students’ feelings of school connectedness significantly declined during the pandemic years (from the 2019–2020 to the 2021–2022 school year), but significantly improved in the 2022–2023 school year, with no significant difference between the pre-pandemic and post-pandemic school years examined [25]. In the 2022–2023 school year, students who had positive relationships with teachers and staff had higher achievement test scores in English Language Arts and Math; however, relationships with teachers were not related to chronic absenteeism. Overall, the U.S. research indicates the likelihood of some changes in students’ mental and behavioral health from before the pandemic to the time when schools reopened in-person, but the intensity, duration, and risk and protective factors associated with this are largely unknown.

In sum, data globally show a greater effect of the pandemic and schools returning to in-person learning on anxiety and depression, compared to other mental health indicators. Relatively few studies reported on changes in externalizing behavior or sleep. Some studies found reductions in mental health symptoms upon school return to in-person learning compared to during the pandemic-related school building closure [17], at least for some age groups [18] but only had data up to a few months post-return to in-person learning. Three studies with pre-pandemic data found that symptoms remained high, even after school returned to in-person learning [19, 20, 23], or increased [21]. This finding demonstrates the need for exploring different student-related and contextual factors that may influence mental health, as well as modifiable risk and protective factors that can help children and youth with long term recovery.

## Risk and Protective Factors in Youth Post-Pandemic Adjustment

Disaster mental health research in general shows that age, gender, and being a part of a minoritized or marginalized group can affect long term post-disaster adjustment [26]. Although the pandemic was longer lasting than most disasters, a disaster mental health framework can help in understanding factors that can affect long term adjustment. For example, understanding demographic trends, especially the possible reasons behind them, can help mental health professionals target resources and supports. This current review on mental health following school building reopening shows that age was associated with some aspects of mental health, but few studies had a comprehensive sample of age ranges to form more definitive conclusions. Depending on the mental health indicator, adolescents often fared worse than younger children [16, 18], but within the subgroup of adolescents, some studies found that the younger adolescents struggled more [19, 22]. For sleep impairment, younger children fared worse in the general population, but among a clinical sample, it was older youth who showed the largest increases in sleep impairment as the pandemic progressed [20]. 

Many of the age-related findings depended on gender and the mental health outcome being measured [19]. The Finnish study found that for younger secondary school girls, the prevalence of depression, generalized and social anxiety symptoms increased from 2021 to 2023, but for older secondary school girls, only social anxiety increased. Among boys, the proportion with social anxiety decreased from 2021 to 2023. Transgender youth had higher rates of generalized anxiety and depression compared to cisgender youth, but the rates for transgender youth decreased from 2021 to 2023. Girls also showed elevated symptoms compared to boys in an Australian study [23]. Within the U.S., YRBS data showed a greater initial decline in mental health for females than males, but also better recovery post-pandemic. These results were similar to the findings from Iceland [22]. However, other studies did not find gender differences [20, 21]. A Canadian study with two time points – one upon school re-opening for in-person learning and the second three-months later – found a decline in mental health among male students in that time span [27], highlighting the need to follow students’ mental health over time to identify patterns of improvement or worsening, even after a return to in-person learning. Taken as a whole, conclusions about the mental health impact of schools returning to in-person learning post-pandemic can depend on the indicator of mental health being observed and the age and gender of the student. Furthermore, particular attention to students with disabilities is warranted [18], and remains understudied. Research suggests that they have higher rates of mental health challenges, in general, and are also more likely to experience other mental health risks (e.g., higher rates of involvement in peer victimization) [28]. 

There are also potentially modifiable mediators and moderators of youth mental health following school return to in-person learning that can be targets of intervention. This includes time spent on homework; [29] internet and social media use; [22, 29, 30] physical activity and time spent in play; [29, 30] communication with others; [29] optimism, connections with others, and pre-pandemic mental health symptoms; [21] positive relationships to teachers and school staff [25], and attitudes towards time spent alone [23]. Within the family, levels of parental support, witnessing parent arguments, and serious arguments with parents [22], as well as parental mental health, hostility and inconsistent discipline [30], all affected youth mental health. Thus, family-focused prevention, early intervention, and treatment options need to continue to be supported and expanded. Finally, addressing sleep impairment, especially for clinical populations, can be critical [20], given its connection to emotion regulation and many different forms of psychopathology.

## Mental Health Supports for Students Post-Pandemic

The COVID-19 pandemic created a massive change in mental health services and their delivery. Within the U.S. context, schools have consistently been a primary setting where youth receive mental health services [31]. Therefore, when schools suspended in-person learning during the pandemic there was concern that many youth would not receive access to their primary mental health support structure. In particular, an increase in the proportion of pediatric emergency department visits for mental health-related reasons raised concern that youth had limited access to the typical avenues for identifying mental health needs and providing ongoing outpatient services [32]. One study examined data from a large academic health system in the U.S. from pre- to during pandemic to understand levels of psychiatric need in the pediatric population. This study found that the average weekly referrals for pediatric behavioral health services increased from pre- to during pandemic, mainly among adolescents, and average weekly outpatient visits increased during the pandemic [33]. However, emergency department and inpatient psychiatry visits decreased, but there were also fewer beds available, perhaps due to physical distancing restrictions [33]. A study of Electronic Health Record data from mid-Atlantic health systems from 2015 to 2023 similarly found an increase in pediatric prescription orders for antianxiety and antidepressant medications [34]. 

Funding for school-based mental health staffing in the U.S. has always come from a complex combination of federal, state, and local sources that vary by district and region [35, 36]. However, during the pandemic existing funding sources were supplemented by Elementary and Secondary School Emergency Relief (ESSER) funds as well as funding from the Bipartisan Safer Communities Act (BSCA), which were designed to address the impact of COVID-19 and gun violence [35, 36]. An estimated $1 billion was allocated by districts to expand school mental health services [35], representing a significant influx of available resources for school mental health services to meet the growing mental health needs of students.

The subsequent expiration of ESSER funds in September of 2024 placed these additional mental health supports at risk of elimination. While the extent to which communities and regions were able to use these funds to establish sustainable infrastructures varied considerably, many schools and communities are again seeking to piece together funding from multiple sources to cover essential school-based mental health services [36]. The rapid and substantial, but temporary, influx of funding for mental health services during the pandemic is similar to other disaster relief systems, though on a much larger scale due to the pervasiveness of the pandemic.

Even before the COVID-19 pandemic, multiple professional organizations reported that most schools did not meet the ideal ratios to be fully staffed with school mental health personnel (e.g., counselors, psychologists; American School Counselor Association; National Association of School Psychologists) [36]. For example, the American School Counselor Association recommends a ratio of 250 students for every 1 school counselor [37]. While the ratio of school counselors to students has improved over time (from 491:1 in 2013–2014 to 376:1 in 2023-24), these ratios still remain well above recommendation [37]. 

Of note, many school districts also contract with community mental health agencies to provide school-based mental health services, which would not be counted in those ratios (numbers reported above only include district employees). Contracting with direct service providers in the community may be a strategic choice compared to hiring a district employee, given the transient nature of ESSER funds, temporary grants, and insurance billing, as these contracts can be terminated or renewed annually based on funding. However, this strategy has the potential disadvantage of engaging mental health staff who are less well integrated in the school community, rely on hourly billing and are therefore less able to engage in collaboration with teachers and other school staff to provide a continuum of services, and have less stability and consistency for students.

Beyond school-based services, the American Academy of Child and Adolescent Psychiatry issued a 2023 policy statement [38] noting the pre-pandemic shortage of mental health professionals of all types in the U.S., with more than half of children with needs not receiving proper behavioral healthcare. The pandemic may have exacerbated this shortage, as demand may have increased, but the shortages themselves are not new.

The U.S. is again facing potential substantial shifts to mental health service coverage. In addition to the increasing mental health needs of youth and diminishment of ESSER funding, proposed changes to Medicaid coverage are likely to impact coverage of services for children and adolescents. To address staffing shortages, some states are developing pathways for bachelor’s level personnel to provide preventive and promotive mental health services. For example, California offers certification as a Wellness Coach [39], a position that can provide wellness promotion and education activities, screening and care coordination, and some types of individual and group support, with the potential to complement or support (though not replace) the work of credentialed school-based mental health providers.

## Implication for Research & Practice

From the extant literature on disaster mental health, we know that for a significant minority of the population, recovery can last for years [26]. The limited available data on trends in mental health post-pandemic suggest ongoing increases in mental health need overall, with some subgroups of youth recovering more rapidly than others. What also seems clear from observations of pandemic response is that the children’s mental health services system has the potential for rapid and substantial shifts in response to large-scale disaster. Changes during the pandemic, including the uptake of telehealth and the considerable influx of relief funding, indicate that large-scale policy and practice changes are possible. What is less clear, however, is whether the same momentum can be leveraged to establish new, innovative, and comprehensive systems of mental health support for youth absent a disaster.

As we remain in the long-term recovery phase following the pandemic, it is critical that we continue to monitor and address child and youth mental health. Schools are a logical point of intervention, since they have access to the general population of children and can be an avenue to address barriers to accessing care [40]. To inform public mental health efforts, we need more studies with longitudinal designs that assess beyond the first few months of school return to in-person learning, and can discern modifiable risk and protective factors affecting trajectories of resilience and recovery. We also need to invest in high-quality studies with representative samples and geographic diversity. Sociodemographic trends are important to consider, but understanding what *mechanisms* may be affecting age, racial/ethnic, or gender-related differences is even more important, as they point to avenues for how to support and effectively use human and financial resources.

## Conclusion

There are a multitude of factors affecting current rates of child and youth mental health problems, including fallout from the pandemic, school return to in-person learning, political stress, the frequency and severity of natural and human-caused disasters, war, and displacement. In addition to their educational functions, schools provide continuity, routine, access to peer and adult support outside the family, time for play and physical activity, and access to school-based mental health professionals. Therefore, we recommend that psychiatrists and other mental health professionals consider the following:

1. Partnering closely with schools and school-based providers to reduce barriers to mental health service access for youth, increase proactive identification and low-intensity supports, and provide coordinated care in response to disasters.
2. Attending to long-term recovery post-disaster, as well as structures and contexts that impact youth recovery over long periods of time. This includes working with communities to ensure sustainable and community-embedded response post-disaster.
3. Actively advocating for disaster recovery resources at the local, state, and national levels, including staffing and funding to be directed to infrastructure supports that may provide lasting mental health care.
4. Continuing surveillance of child and youth mental health post-pandemic, as children who were ages birth to age 4 in the pandemic are now entering school, and it is unclear how pandemic losses or public-health restrictions affected their social emotional development and subsequent mental health. Ultimately, ongoing surveillance will lead to the most important and actionable information for schools and communities to determine who needs help and what types of support are most effective.

## Data Availability

No datasets were generated or analysed during the current study.
